# Human mobile robot interaction in the retail environment

**DOI:** 10.1038/s41597-022-01802-8

**Published:** 2022-11-04

**Authors:** Yuhao Chen, Yue Luo, Chizhao Yang, Mustafa Ozkan Yerebakan, Shuai Hao, Nicolas Grimaldi, Song Li, Read Hayes, Boyi Hu

**Affiliations:** 1 Department of Industrial and Systems Engineering, University of Florida, Gainesville, FL 32611 USA; 2 Department of Mechanical and Aerospace Engineering, West Virginia University, Morgantown, WV 26506 USA; 3 J. Crayton Pruitt Family Department of Biomedical Engineering, University of Florida, Gainesville, FL 32611 USA; 4 Department of Computer and Information Science and Engineering, University of Florida, Gainesville, FL 32611 USA; 5 Herbert Wertheim College of Engineering FLEX, University of Florida, Gainesville, FL 32611 USA; 6 Loss Prevention Research Council, Gainesville, FL 32611 USA

**Keywords:** Social sciences, Scientific community, Engineering

## Abstract

As technology advances, Human-Robot Interaction (HRI) is boosting overall system efficiency and productivity. However, allowing robots to be present closely with humans will inevitably put higher demands on precise human motion tracking and prediction. Datasets that contain both humans and robots operating in the shared space are receiving growing attention as they may facilitate a variety of robotics and human-systems research. Datasets that track HRI with rich information other than video images during daily activities are rarely seen. In this paper, we introduce a novel dataset that focuses on social navigation between humans and robots in a future-oriented Wholesale and Retail Trade (WRT) environment (https://uf-retail-cobot-dataset.github.io/). Eight participants performed the tasks that are commonly undertaken by consumers and retail workers. More than 260 minutes of data were collected, including robot and human trajectories, human full-body motion capture, eye gaze directions, and other contextual information. Comprehensive descriptions of each category of data stream, as well as potential use cases are included. Furthermore, analysis with multiple data sources and future directions are discussed.

## Abstract (summary)

**Table:** 

Measurement(s)	Robot and human trajectories, human full-body motion capture, and eye gaze directions.
Technology Type(s)	Mobile robot, inertial measurement unit-based motion capture system, and eye tracker.

## Background & Summary

Utilizing artificial intelligence (AI) methods to accurately model human motion patterns (trajectory, posture, etc.) has received substantial attention recently. This direction of research has broad implications in many domains, such as autonomous driving^1–3^, collaborative robots (co-bots)^4–7^, and public safety surveillance^8,9^. Over many millennia, our ancestors have acquired the skill to incorporate the intentions and responses of other nearby agents (e.g., humans, pets, vehicles) into their decision making process. This resilient survival and social skill has quietly supported many aspects of our modern life, from navigating through crowded spaces to driving on a local street or merely acting civil among other people^10^. In order to operate safely and appropriately in dynamic and complex environments, robots, like humans, should employ accurate human motion perception and human-like behavior planning as critical ‘awareness’ and decision-making capabilities, especially when sharing space with humans.

The era of ubiquitous co-bots is becoming a reality with the rapid advancements of AI, robotics, control, 5G, and many other enabling techniques. During the COVID-19 pandemic, co-bots were able to interact with humans in close quarters in many environments to promote social distancing. Tasks performed included surveillance^11^, administering COVID-19 tests^12^, and disinfecting ground surfaces^13^. This utility has proven vital during the pandemic as physical contact became more dangerous and less desirable. Even after the pandemic ends, these co-bots are unlikely to disappear as people have begun to see the utility of robots, which will lead to higher acceptability^14^. It would be the new norm that humans and robots share the same space and collaborate closely. To achieve seamless and harmonic HRI, robots need to perceive human intention precisely and behave in a socially compatible manner. One particular bottleneck is the scarcity of publicly accessible datasets of HRI that AI models can learn from. Datasets that include HRI scenarios are even rarer and the contextual information aspect of them can be further improved, such as high-fidelity simulation environments, and detailed human participants’ intention and motion behaviors. Several human trajectory datasets have been published. Many of them, such as ETH^15^, UCY^16^, Edinburgh^17^, Town Center^18^, and Daimler Pedestrian^19^, were captured outdoors using one or more video cameras. There are also datasets collected indoors, such as JRDB^20^, Central station^21^, ATC^22^ and MoGaze^23^. However, most of these datasets have only annotated humans with bounding boxes, neglecting the merits of other contextual information such as human intention, body posture, and joint level kinematics. Furthermore, they did not include detailed human physical locomotion as well as physiological performance during the tasks. There are many datasets devoted to monitoring and recording human movements via various motion capture systems. Examples such as CMU Graphics Lab Motion Capture Database^24^, Human3.6 m^25^, and KIT^26^ were recorded using the optical motion capture system (e.g., Vicon), whereas datasets from^27,28^, and^29^ were recorded using wearable sensors. However, these datasets are mostly restricted to standalone (lack of interactions between humans and surrounding environment and agents) and domestic daily activities (e.g., sitting, standing). A limited number of datasets can be found that cover human and man-made agent interactions. Datasets include HRI are even rarer. Table 1 summarized six publicly accessible datasets that include diversified agents in the environment. Three datasets, L-CAS, KTH, and THÖR include one or more robots. Among them, only THÖR contains detailed contextual information such as human intention and human body postures. The lack of such datasets became the primary motivation for this research activity.

**Table 1:** A summary of publicly accessible datasets that include human and other agents in the data collection environment.

Dateset	Author & Year	Environment	Agents			Systems			
			Robot(s)	Human	Vehicle(s)	ET*	PM*	HM*	VD*
Stanford Drone	Robicquet *et al*., 2016^45^	Outdoor		✓	✓				✓
VIRAT	Oh *et al*. 2011^46^	Outdoor		✓	✓				✓
KITTI	Geiger *et al*. 2012^47^	Outdoor		✓	✓				✓
L-CAS	Yan *et al*. 2017^48^	Indoor	✓	✓					✓
KTH	Dondrup *et al*. 2015^49^	Indoor	✓	✓					✓
THÖR	Rudenko *et al*. 2020^40^	Indoor	✓	✓		✓		✓	✓

*ET: Eye-Tracking; PM: Physiological Measurement; HM: Human Motion; VD: Video.

Therefore, more comprehensive datasets are required to foster future progress in robot learning. In this paper, we introduce a dataset that focuses on social navigation between humans and robots in a retail environment. The WRT domain is selected as the focus and test-bed of this work because of the significant increase in robot usage in this industry sector. The usage of robots in the WRT sector has been amplified by the emergence of the pandemic which has prompted a crucial need for the safe integration of this technology into the industry. Furthermore, compared to other common robot deployment environments (manufacturing plants, large scale distribution centers, etc.), retail settings present more direct and frequent HRI opportunities, which makes human motion data more valuable.

## Methods

### Participants

The data collection was done with eight healthy participants, five males and three females. All participants were recruited from the University of Florida student population and reported being healthy. Their mean (SD) age, height with shoes on, and body weight were 19.4 (2.0) years old, 176.7 (10.2) cm, and 66.0 (10.1) kg. Seven of the participants were self-reported to be right-handed and one was reported to be ambidextrous. Participants voluntarily agreed to be recorded and were informed that the data collected in the study will be made public. The experiment protocol was approved by the University of Florida Institutional Review Board (IRB202002765).

### Test environment setup and instruments

**Fig. 1:**
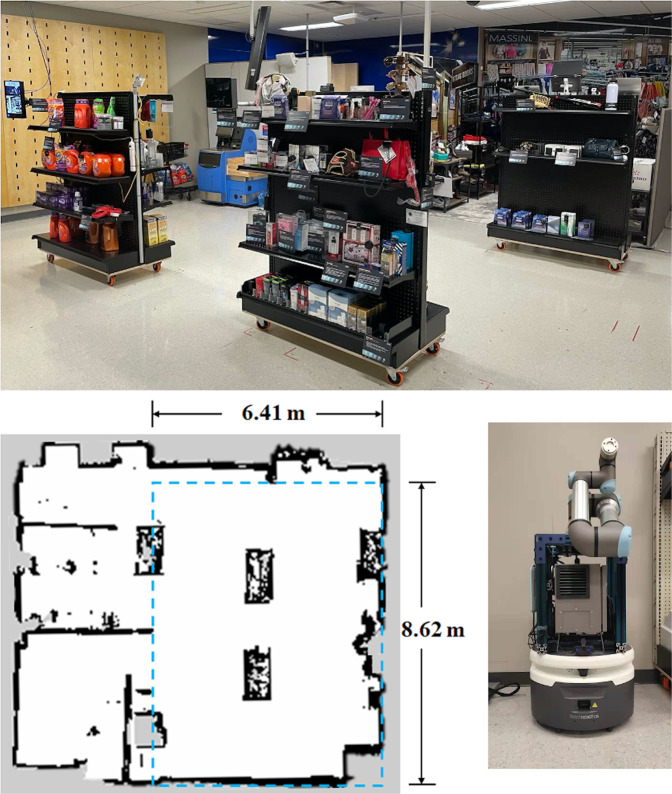
Top: a picture of the future-oriented WRT research facility. Bottom Left: 2D costmap of the facility and dimensions of the experiment area. Bottom Right: a picture of the customized mobile robot platform used in the experiment.

The experiment was conducted in a future-oriented WRT research facility (Fig. 1). This laboratory allows researchers and practitioners to conduct studies and practice interaction protocols with emerging WRT methods. These include no/low touch locking systems, anti-theft devices, protective displays, day/night cameras with edge-AI, and special public view monitors that provide personalized advertising or messaging. It also includes configurable walls and shelves, and multi-functional units, enabling a highly flexible physical layout adjustment capability.

The robot used in the experiment was a customized mobile robot platform consisting of a Fetch Freight Base (Fetch Robotics, Inc., San Jose, California) and a UR5 robot manipulator (Universal Robots, Odense, Denmark). The robot platform has a footprint of 0.508 × 0.559 m and a height of 1.295 m, as shown in Fig. 1. The mobile robot base was operated in Robot Operating System (ROS) with an Intel i3 processor, 8 GB RAM, and 120 GB SSD. The UR5 manipulator was 18.4 kg in weight and it has six rotating joints and a maximum payload of 5 kg. The robot platform was equipped with an embedded 2D Lidar, which has a scanning range of 25 m and 220 degrees field of view (TIM571 SICK, Waldkirch, Germany), a Logitech C920 webcam (Logitech, Lausanne, Switzerland), a 6D Inertial Measurement Unit (IMU), and two wheel encoders. By integrating the webcam on the mobile robot, the video data was saved in the rosbag format along with other robot data. Furthermore, the videos were automatically synchronized with other robot data, as opposed to a stand-alone camera on the ceiling. During the experiment, the maximum speed of the robot was set at 1.0 m/s and the UR5 was powered off and remained retracted.

The same mobile robot control scheme was employed as in our earlier studies^30,31^. Briefly, the control scheme was developed based on a 2D navigation framework^32^, which includes functional modules for localization, global path planning, local path planning (i.e., collision avoidance), and robot movement control. The costmap (i.e., occupancy grid map) of the environment was pre-generated using the Lidar-based gmapping technique^33^ since layouts in WRT environments are often organized and consistent. The Monte Carlo localization approach^34^ was used to localize the robot using 2D Lidar and embedded inertial sensors and wheel odometry. This method compares Lidar scans to the previously acquired map using a particle filter to estimate the robot’s pose. The Dijkstra’s algorithm^35^ was adopted to determine the global traverse path on the known map. The Trajectory Rollout algorithm^36^, a widely applied and effective collision avoidance algorithm, was used as our local path planner. The robot was then guided along the local and global path using a velocity-based proportional controller.

To avoid the marker occlusion problem that is presented in camera-based motion capture (MOCAP) systems, Xsens (MVN Awinda, Xsens Technologies BV, Enschede, Netherlands), an IMU-based MOCAP system, was chosen for the study. The use of IMU sensors with full-body configuration enriched the dataset with human localization and body posture information. The sampling frequency of the MOCAP system was set at 60 Hz throughout the project.

In order to acquire eye gaze data, a pair of Tobii Pro Glasses 2 (Tobii, Danderyd Municipality, Sweden) was worn by participants with appropriate corrective snap-on lenses if needed. The sampling frequency of the eye-tracker was set at 50 Hz. In addition to the eye gaze data, the eye-tracker also contained a scene camera that recorded 1920 × 1080 video at 25 fps.

### Tasks and procedure

**Fig. 2:**
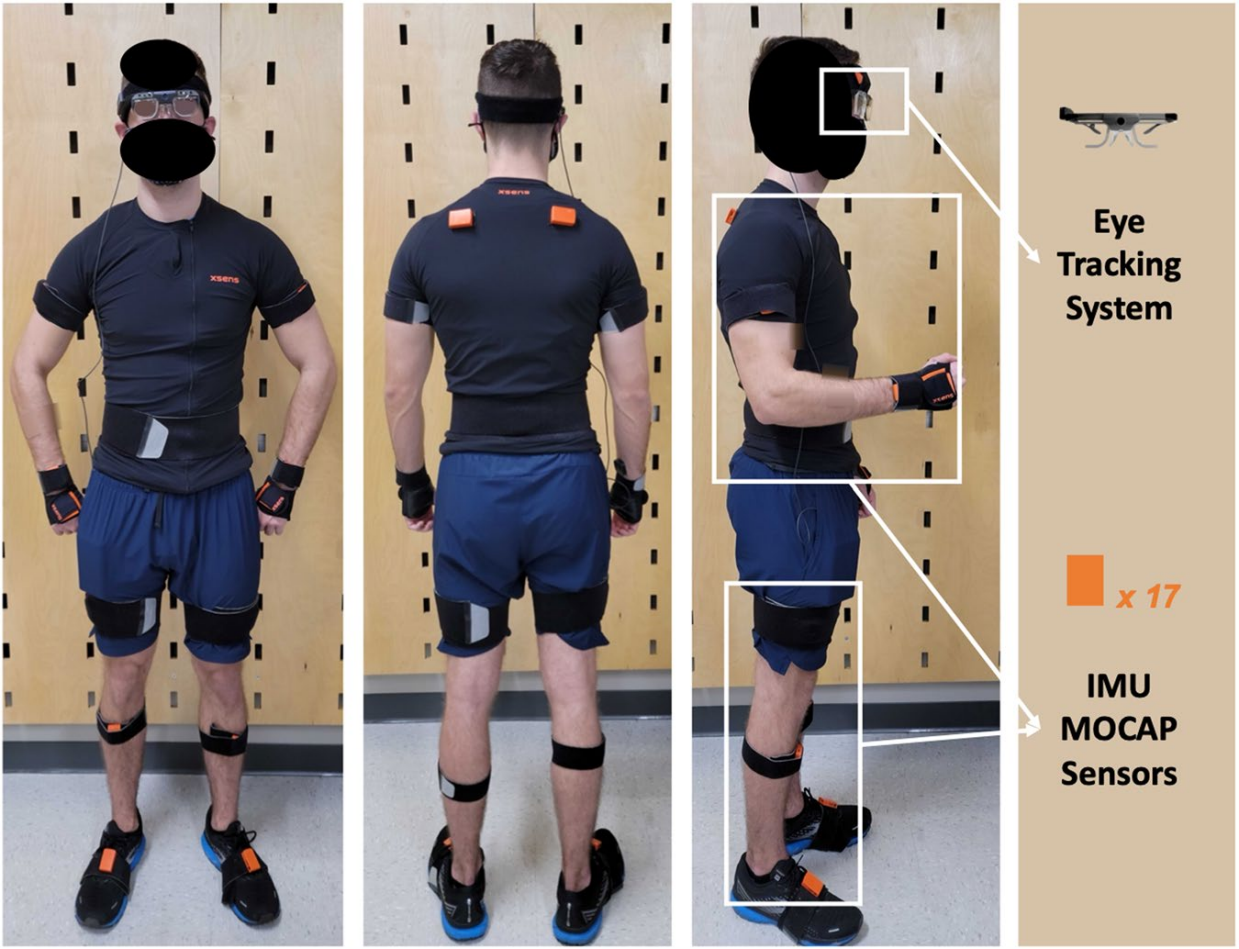
Sensor setup for the experiment. The participant consented for the publication to utilize their likenesses.

Table 2 summarizes the main tasks involved in the data collection and their approximate durations. Right after the arrival, each participant received instructions for the test environment setup and instruments. Questions and concerns were addressed at the same time. Subsequently, demographic data including age, gender, weight, and height with shoes on were obtained. Next, 17 IMU sensors were securely attached to participants’ body, specifically on: head, sternum, pelvis, right and left shoulder, right and left upper arm, right and left forearm, right and left hand, right and left upper leg, right and left lower leg, right and left foot^37,38^ (Fig. 2). The MOCAP system was then calibrated based on each participant’s body dimensions and calibration activities. A total of twelve body dimension measures were included: body height, foot or shoe length, shoulder height, shoulder width, elbow span, wrist span, arm span, hip height, hip width, knee height, ankle height, extra shoe sole thickness. Two calibration activities were: N-pose standing and straight-line walking. Following the MOCAP calibration, the eye-tracker was mounted to the participant’s head as shown in Fig. 2. After the sensor attachment and system calibration had been completed, participants were given two tasks to perform. These tasks are commonly undertaken by WRT consumers and workers, as stated below.

**Table 2:** Main tasks involved in the data collection and their approximate durations.

Task order	Task description	Approximate duration
1	Introduction of environment and instrument.	5 mins
2	Gathering demographic information.	2 mins
3	IMU MOCAP sensors attachment.	10 mins
4	MOCAP system calibration.	5 mins
5	Eye-tracker placement.	2 mins
6	Eye-tracking system calibration.	1 min
7	Introduction of the tasks.	5 mins
8	Ordering picking and sorting trials.	2 mins × 10 repetitions = 20 mins
9	Inventory checking trials.	3 mins × 4 repetitions = 12 mins

#### Task I - Order picking and sorting

**Fig. 3:**
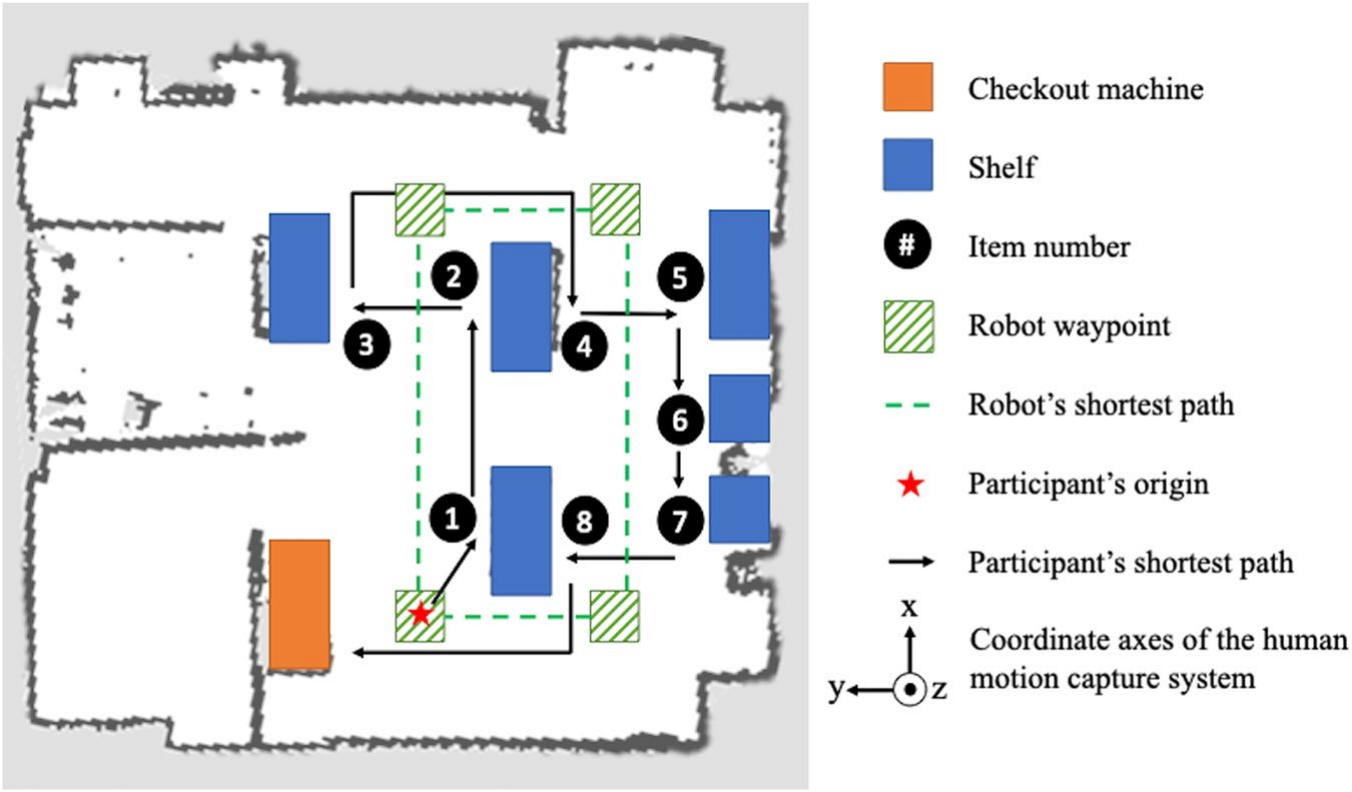
Demonstration of the ordering picking and sorting task in the WRT facility along with the predefined waypoints of the robot. The origin and axis directions of the human motion capture system are also included.

For this task, the participant was asked to push a shopping cart and retrieve eight different items, one from each of the eight shelves. Afterwards, the participant returned to the self-checkout machine to sort the first four items into one bin and the other four into another bin. The shopping cart itself was 37 lbs in weight, and in order to simulate real-world conditions, its weight was added to 100 lbs and controlled to be the same for all participants. There were two different conditions of the task: 1) picking and sorting alongside the robot, and 2) picking and sorting without the robot. Each condition had five trials (i.e., 2 × 5 = 10 trials of order picking and sorting). The repetition for each condition was determined to prevent any fatigue (mental or physical) accumulated from the test. The participant experienced five repetitions of one condition then the other five. In each trial, the participant was given a list showing the items that they were required to pick up; the item on each shelf was randomly selected. The same five lists were used in both conditions. The participant was asked to pick the items in order and use their dominant hand to perform the task. This included picking, sorting, and cart pushing (whenever one hand cart pushing is needed). No training was given prior to the data collection. The co-bot in the current study represented a generic mobile platform which can mock the motion trajectory of multiple functions in retail environments such as disinfection, cleaning, and inventory management. The robot in the experiment was programmed to move between waypoints automatically and it was able to avoid obstacles and replan routes. The waypoints were predefined so that the participant and the robot came across each other frequently (Fig. 3). In order to ensure that no physical collision would happen during the trials, the researcher assumed control of the robot when necessary. These instances were marked as “intervened” in the data sheets.

#### Task II - Inventory checking

**Fig. 4:**
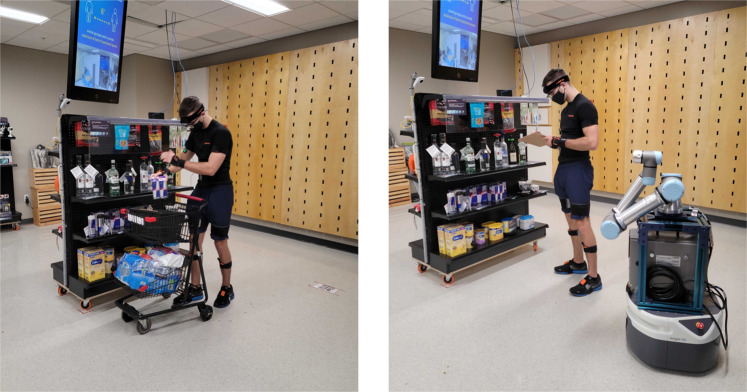
Left: An example picture of the participant picking an item from the shelf. Right: The participant performed the inventory checking task while the robot went by. The participant consented for the publication to utilize their likenesses.

Subsequent to the order picking and sorting tasks, four inventory checking trials were performed by each participant. Similar to task I, there were also two conditions, i.e., inventory checking alongside the robot and inventory checking without the robot. In each trial, the participant was given a checklist showing the items that need to be checked from the eight shelves (Fig. 4). The participant had to count the items on the list in order. The shopping cart was not utilized at any point throughout this task. The participant either experienced the robot condition or the non-robot condition first, and each condition had two repetitions (i.e., 2 × 2 = 4 trials of inventory checking). Items from each shelf were randomly selected and the same two checklists were used in both conditions. No training was given prior to this task.

It’s worth noting that the task selection was determined after careful consideration. The main challenge in the HRI domain comes from the ‘interaction’, ‘collaboration’, and ‘cooperation’ between agents (both physically and cognitively), especially for HRI scenarios with fixed manipulators. However, regarding mobile robot applications, due to their advanced mobility in the spatial domain, interactions between mobile robots and human partners may occur in a much larger space (spatial) or in different forms (temporal). Specifically, in the retail environment, the direct interactions between human and robot are still not commonly seen other than a few exploratory applications like auto shopping guides or shopping carts. Most of the current adopted retail co-bots do not interact directly with humans, such as floor cleaning robots, disinfection robots, and inventory scanning robots. However, they still interact with each other in a more general sense and do not merely co-exist in the same space with human partners. For example, the mobile robot may be auto floor cleaning robot or an inventory scanning robot, their behavior will inevitably influence the shopping experience of human partners substantially and the overall ecosystem (i.e. retail store) performance. To ensure good practicability, the current work included testing scenarios involving these more commonly seen applications, instead of those ones that are still further beyond the horizon. Investigations of the selected HRI scenarios in the retail environment may lead to examining how humans respond physically and cognitively to mobile robots and developing more socially aware robot motions.

## Data Records

More than 260 minutes of data were recorded, including data of robot sensors, human motion capture, and eye gaze measurements. In order to provide free accessibility to the public, the data has been uploaded to Science Data Bank^39^ (10.11922/sciencedb.01351), an open generalist data repository developed and maintained by the Computer Network Information Center of the Chinese Academy of Sciences. Readers can also access the dataset through the website (https://uf-retail-cobot-dataset.github.io/), where a detailed description of each type of data is available. Among 112 trials accomplished by eight participants, 80 (8 participants × 5 repetitions × 2 conditions) of them were ordering picking and sorting tasks, and inventory checking tasks accounted for the other 32 (8 participants × 2 repetitions × 2 conditions) trials. The details of recorded data are listed as follows.

### Robot sensor data

All sensor data from the robot, including images from camera, point clouds from Lidar, measurements of acceleration and angular rate from IMU, and joint states from wheel encoders, were recorded as the serialized ROS message data in the rosbag format. Note that, in order to perform online recordings, the images were recorded as 640 by 480p, which is on par with comparable works and is sufficient to show the information from the first-person robot view. Moreover, the navigation information (i.e., the online pose estimates and planned path) during each trial, which will be discussed in the following analysis, was also recorded in the rosbag files. Since only half of the trials were robot involved, 56 (40 order picking and sorting + 16 inventory checking) bag files are available in the dataset.

### Human motion capture

A total of 112 excel files are available in the MOCAP data folder. Four trials were found to have poor data quality (i.e., off-axis), and the corresponding files were labeled as “offaxis”. The human motion data from Xsens contains over 930 thousand frames of human posture information (i.e., joint positions). The following human motions were observed frequently during the experiment: 1) picking up and putting down the item list, 2) pushing and pulling the shopping cart, 3) browsing through the shelf, 4) bending over and crouching, 5) picking up items from the shelf, 6) putting items into the bin, 7) writing on the checklist, 8) walking between shelves, 9) counting items, and 10) avoiding robot if necessary. With video recordings and wearable motion tracking data stream (all are accessible), this semantic information can be extracted and labeled by the public. Inside each participant folder, trial files contain the timestamp information and position data of 23 joints, including pelvis, L5, L3, T12, T8, neck, head, right and left shoulder, right and left upper arm, right and left forearm, right and left hand, right and left upper leg, right and left lower leg, right and left foot, right and left toe. All position data use the same coordination system (i.e., the origin and axis directions), as shown in Fig. 3. In addition, participant identification number (from 1 to 8) and trial identification number (from 1 to 14) were properly marked.

### Eye-tracking data

Eye-tracking data, such as gaze directions and eye movements, were saved in 112 excel files (8 participants × 14 trials). The collected data were exported with timestamps so that they can be further analyzed along with other measurements. In addition, a total of 112 videos (.MP4) recorded by the embedded scene camera are also available in the current dataset.

### Participant data sheet

Participants’ demographic data, trial conditions, and task durations were recorded in “Participant data sheet.docx”.

## Technical Validation

### Sensor placement

Participants were required to wear tight clothes during the experiment to prevent sensor movement. As described in the Methods section, the placement of wearable sensors (i.e., IMU sensors and eye-tracker) was according to the manufacturer’s instructions. In addition, before each experiment trial, the signal quality of each sensor was manually verified through the system’s acquisition software. Furthermore, the wearable sensors were attached by the same researchers for consistency.

### Evaluation metrics of human trajectories

To evaluate the quality of human trajectories recorded in the current dataset, methods proposed by Rudenko *et al*.^40^ were used to compute the tracking duration, trajectory curvature, perception noise, and motion speed of each trial. The position of the IMU sensor on the pelvis was used for the calculation as it is nearly equivalent to the human body’s center of mass. All position data were filtered by a low-pass filter (Butterworth, 2nd order, 6 Hz cut-off frequency, zero lag) before the calculation. Results were compared with existing relevant datasets: THÖR^40^, ETH^15^, ATC^22^, and Edinburgh^17^. As shown in Table 3, our dataset has a longer duration (143.7 ± 53.8 s) and a higher trajectory curvature (13.5 ± 39.7 m^−1^) when compared to other datasets. This indicates that the walking paths traversed by our participants are relatively more complicated and non-linear. In addition, a relatively high perception noise (0.65 m⋅s^−2^) and slow motion speed (0.21 ± 0.25 m⋅s^−1^) were observed in the current dataset, which could be attributed to participants’ extended static period during the item searching phase of the task. The mixture of activities (e.g., walking and standing) in such a confined space with the presence of a mobile co-bot close by potentially further increased the trajectory curvature and perception noise, while lowering the overall motion speed as compared to simpler tasks. Overall, these analyses collectively validate the unique value our dataset can bring to the research community.

**Table 3:** Comparison of datasets (results of the existing datasets were retrieved from Rudenko *et al*.^40^).

Metric	The Current Study	THÖR	ETH	ATC	Edinburgh
Tracking duration (s)	143.7 ± 53.8	16.7 ± 14.9	9.4 ± 5.4	39.7 ± 64.7	10.1 ± 12.7
Trajectory curvature (m^−1^)	13.5 ± 39.7	1.9 ± 8.8	0.2 ± 1.5	0.8 ± 1.4	1.0 ± 3.9
Perception noise (m⋅s^−2^)	0.65	0.12	0.19	0.48	0.81
Motion speed (m⋅s^−1^)	0.21 ± 0.25	0.81 ± 0.49	1.38 ± 0.46	1.04 ± 0.46	1.00 ± 0.64

## Usage Notes

### Machine learning model training

**Fig. 5:**
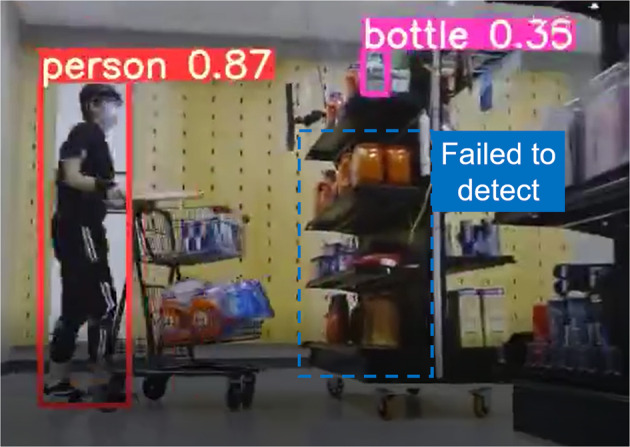
A demonstration figure of the video frame annotated by YOLO V5. The participant consented for the publication to utilize their likenesses.

The video camera on the mobile robot recorded the surrounding environment, which can be used to train varied computer vision based Machine Learning (ML) models. As a proof of concept, we deployed the YOLO V5^41^, a state-of-the-art object detection architecture pre-trained on COCO dataset, on our video images. Results are interesting: pre-trained models can reliably detect the human worker and large objects in the scene with relatively high confidence. In terms of the items and products on the shelves, however, there is a considerable amount of false detection and failed detection (Fig. 5). To address the above problem and further fine-tune the ML models, more contextual based high quality video images are necessary. We hope that our initial efforts will result in  the emergence of a new wave of appropriate datasets.

### Comparison of human trajectories between tasks and conditions

**Fig. 6:**
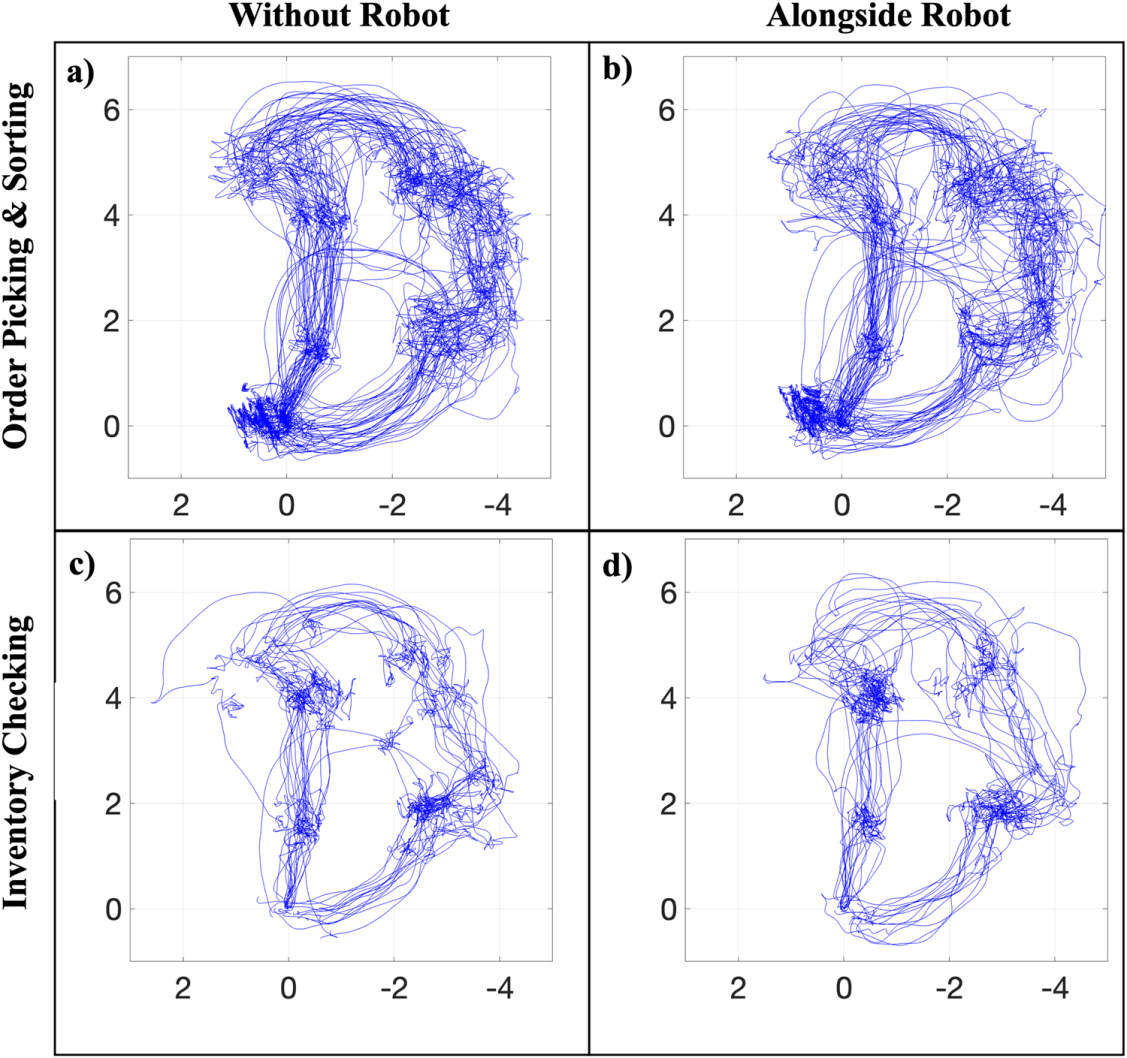
Participants’ trajectories under two robot conditions (without robot vs. alongside robot) during the order picking and sorting task and the inventory checking task.

Participants’ trajectories under two robot conditions (without robot vs. alongside robot) are plotted in Fig. 6 to show the deviation participants made during each task (e.g., order picking and sorting task and inventory checking task). In contrast to those tasks without the robot (Fig. 6a), participant trajectories during order picking and sorting alongside the robot (Fig. 6b) presented a more variant and deviant pattern. During inventory checking jobs, there is no appreciable variation in the trajectory patterns with or without the robot present. Meanwhile, there is no discernible difference in the trajectory patterns between the alongside and without robot conditions during inventory checking tasks. The extended duration of static standing required for inventory checking tasks may be one explanation for why no difference was found. It is possible that during the inventory checking tasks, the majority of potential human-robot collisions were avoided by the moving robot adjusting its trajectory rather than by the standing participants. Future follow up analysis is required to further confirm the rationale and uncover any intriguing behavior responses of participants when interacting with a robot.

### Robot’s trajectory, participant’s trajectory, eye-tracking, and MOCAP data fusion

**Fig. 7:**
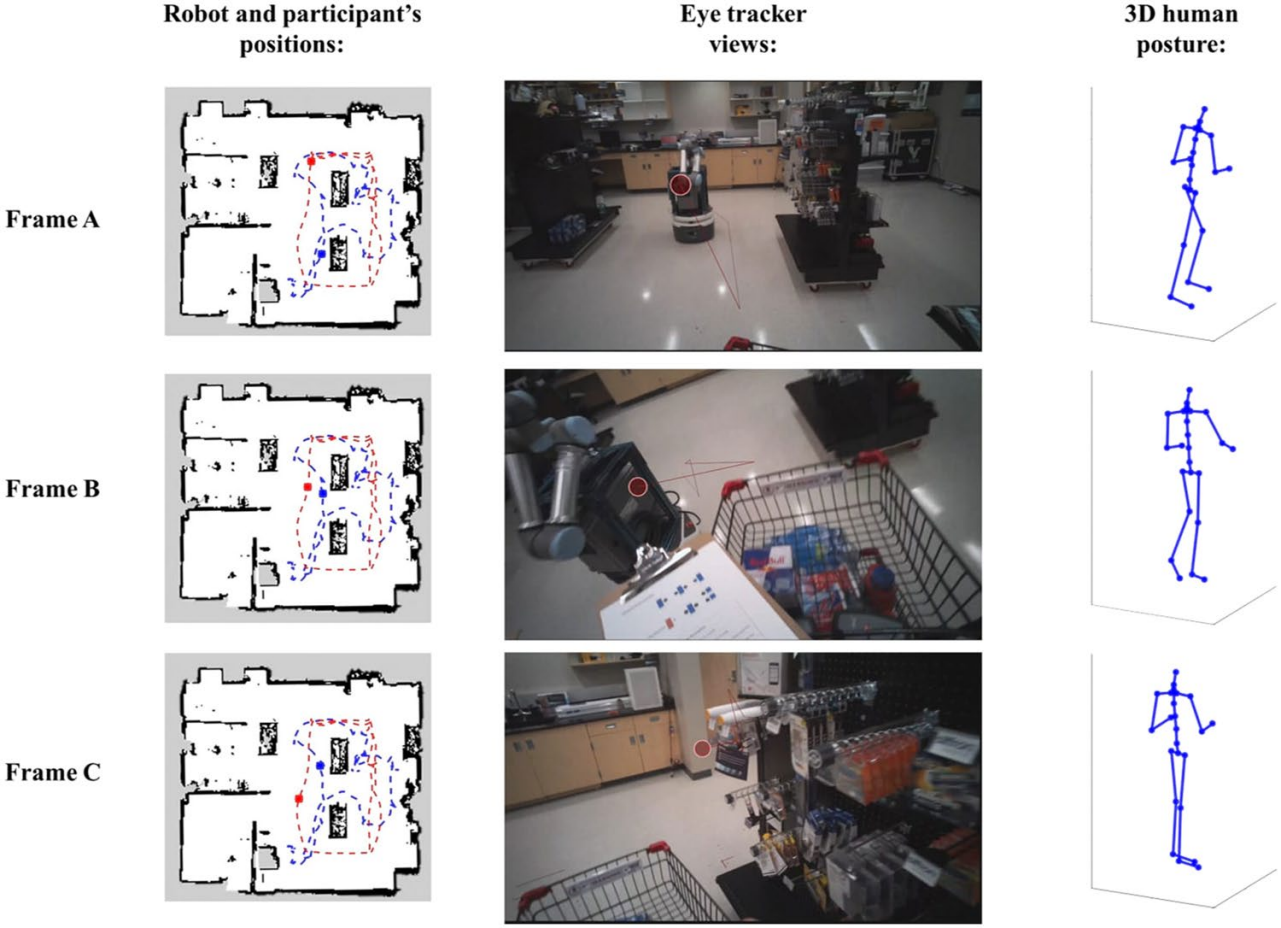
Images on the left column show the mapping of human and robot positions at different time frames in the trial. In the middle are the screenshots from the eye-tracker at the corresponding frames. On the right column are the figures of the 3D human model created with Xsens MOCAP data. Frame A represents the participant’s first glance at the robot. Frame B represents the moment when the participant and the robot begin to avoid each other. Frame C shows the participant following the avoidance interaction, looking for an item on the shelf.

By integrating outputs from both the robot and human motion tracking instrument, it was possible to investigate their collective motion patterns. Furthermore, by using the extra data dimension of the human physiological measurement, a more detailed and precise spatial-temporal pattern analysis was able to be obtained. Figure 7 shows the integration of robot and human positions, eye-tracking information, as well as human 3D posture from one randomly selected sample trial (P001_06). The robot’s positions (red dashed line) were estimated using the adaptive Monte Carlo localization algorithm, which uses a particle filter to track the position of a robot through matching the point clouds to a known map^42^. The map of the retail environment was constructed by the robot prior to the experiment using lidar based gmapping approach^33^. Pelvis 2D positions extracted from the MOCAP data were used to determine the participant’s positions (blue dashed line).

Trajectories of both robot and participant were then overlaid on the map of the retail environment. In addition, by synchronizing the position data with the eye-tracking system using the timestamp, human and robot behaviors (e.g., obstacle avoidance) can be better visualized and investigated. The participant’s full-body posture at different time frames were also demonstrated using the 3D position data of 23 segments. In brief, this sample piece of data depicts a complete scene of a human participant meeting and avoiding a co-bot. The scene begins with the participant virtually identifying the robot, followed by a sequence of mental decision making and physical maneuvers to deviate from the previous trajectory and avoid a potential collision, and ends when the two agents successfully departed and returned back to their normal working modes. Our dataset will be valuable for roboticists to better design safe robot control strategies in human populated environments, especially in safety critical scenarios^31^, as well as for behavior scientists and human-systems researchers to better understand the human fundamental behavior when interacting with robots. For future work, in addition to the robot control scheme adopted in this study, we plan to test multiple robot navigation algorithms and collision avoidance strategies, such as Fuzzy control based navigation algorithms^43^, reinforcement learning based robotic navigation algorithms^44^. It would be interesting to investigate how effectively the robots operate and how people respond to them in a shared space.

## Data Availability

The following GitHub repository contains the custom MATLAB script (R2020a) for loading and visualizing robot trajectory and motion capture data shown in Fig. 7: https://github.com/UF-ISE-HSE/UF-Retail-HRI-Dataset.
